# Automated Diagnosis and Assessment of Cardiac Structural Alteration in Hypertension Ultrasound Images

**DOI:** 10.1155/2022/5616939

**Published:** 2022-05-29

**Authors:** U. Raghavendra, Joel Koh En Wei, Anjan Gudigar, Akanksha Shetty, Jyothi Samanth, Ganesh Paramasivam, Sujay Jagadish, Nahrizul Adib Kadri, Murat Karabatak, Özal Yildirim, N. Arunkumar, Ali Abbasian Ardakani

**Affiliations:** ^1^Department of Instrumentation and Control Engineering, Manipal Institute of Technology, Manipal Academy of Higher Education, Manipal 576104, India; ^2^Department of Electronics and Computer Engineering, Ngee Ann Polytechnic, Clementi 599489, Singapore 599489, Singapore; ^3^Department of Cardiovascular Technology, Manipal College of Health Professions, Manipal Academy of Higher Education, Manipal 576104, India; ^4^Department of Cardiology, Kasturba Medical College and Hospital, Manipal Academy of Higher Education, Manipal 576104, India; ^5^Department of Biomedical Engineering, Faculty of Engineering, University of Malaya, Kuala Lumpur 50603, Malaysia; ^6^Department of Software Engineering, Firat University, Elazig, Turkey; ^7^Department of Biomedical Engineering, Rathinam College of Engineering, Coimbatore, India; ^8^Department of Radiology Technology, School of Allied Medical Sciences, Shahid Beheshti University of Medical Sciences, Tehran, Iran

## Abstract

Hypertension (HTN) is a major risk factor for cardiovascular diseases. At least 45% of deaths due to heart disease and 51% of deaths due to stroke are the result of hypertension. According to research on the prevalence and absolute burden of HTN in India, HTN positively correlated with age and was present in 20.6% of men and 20.9% of women. It was estimated that this trend will increase to 22.9% and 23.6% for men and women, respectively, by 2025. Controlling blood pressure is therefore important to lower both morbidity and mortality. Computer-aided diagnosis (CAD) is a noninvasive technique which can determine subtle myocardial structural changes at an early stage. In this work, we show how a multi-resolution analysis-based CAD system can be utilized for the detection of early HTN-induced left ventricular heart muscle changes with the help of ultrasound imaging. Firstly, features were extracted from the ultrasound imagery, and then the feature dimensions were reduced using a locality sensitive discriminant analysis (LSDA). The decision tree classifier with contourlet and shearlet transform features was later employed for improved performance and maximized accuracy using only two features. The developed model is applicable for the evaluation of cardiac structural alteration in HTN and can be used as a standalone tool in hospitals and polyclinics.

## 1. Introduction

Hypertension (HTN) is a risk factor that is responsible for the increasing incidence of cardiovascular diseases and can increase their morbidity and mortality. HTN, when uncontrolled, may contribute to coronary artery disease, left ventricular hypertrophy (LVH), arrhythmias, hemorrhagic/ischemic stroke, and heart failure. According to recent WHO reports [1], the estimated global prevalence of HTN is 26%, and it is expected to rise to 29% by 2025, expressed mainly in developing countries. According to multiple epidemiological reports, in India, 25–30% of the urban population and 10–20% of rural adults suffer from HTN [2–4].

The heart pumps blood cyclically into the arterial circulation. In HTN, increased afterload causes the heart to work harder in order to pump blood in the forward direction (https://www.who.int/cardiovascular_diseases/publications/global_brief_hypertension/en/). This induces the heart muscle to grow thicker, or hypertrophy. Over time, when uncontrolled, the changes become progressively severe and can lead to heart failure. Increase in left ventricular muscle wall thickness (i.e., LVH) can be objectively quantitated in advanced stages using noninvasive cardiac imaging (e.g., echocardiography). However, echocardiographic assessment is neither sensitive nor specific for identification of early HTN-induced changes prior to hypertrophy. Furthermore, the adaptive mechanism in high blood pressure results not only in LVH but also in atherosclerosis. There may be a latent course in which pathophysiological changes occur prior to the manifestation of primary HTN [5]. This explains the presence of subclinical cardiovascular pathology during disease progression. In this regard, our study, described herein, was designed to evaluate the underlying pathognomonic features among newly diagnosed HTN patients, irrespective of the presence or absence of LVH, using echocardiographic images with a computer-aided detection (CAD) module. We hypothesized that HTN may induce subtle structural changes in the heart muscle that are discernible with intelligent systems. We then designed and developed such a system to discern HPT versus normal subjects using echocardiographic images.

According to recent American College of Cardiology (ACC) and American Heart Association (AHA) guidelines for the detection, prevention, management, and treatment of high blood pressure, stage one HTN is defined as a systolic blood pressure (SBP) of 130–139 mmHg and/or diastolic blood pressure (DBP) of 80–89 mmHg. Stage 2 HTN is diagnosed when the SBP rises above 140 mmHg and/or the DBP is at least 90 mmHg. An SBP over 180 mmHg or DBP above 120 mmHg or both indicate the need for rapid change in medication in the absence of contraindications, or hospitalization if signs of organ damage are present. Using echocardiographic based CAD to diagnose the early changes will enable the detection of nascent HTN-related muscle remodeling, so that blood pressure lowering medications can be promptly instituted to arrest the progression of hypertensive heart disease and its complications.

Currently, CAD has become a commonly utilized technique for clinical diagnostics using ultrasound images [6–10]). It is noted from the literature that several studies on pulmonary hypertension had been made [11–13]. Albà et al. [13] have proposed a CAD tool to accelerate the screening of pulmonary hypertension. During the construction of the automated tool, for training and testing, they used 98 subjects, 49 normal and 49 abnormal. Albà et al. have also proposed a globally weighted local binary pattern for quantitative analysis, achieving 92% accuracy and 96% sensitivity. In [14], several machine learning algorithms to detect hypertension are reviewed. Herein, the authors have performed hypertensive detection using clinical and socio-demographic data. The increased blood pressure is predicted using tree classifier and compared with logistic regression (LR) [15]. To know whether a person suffered from hypertension or not, the authors have used logistic regression model [16]. It is also noted that, using annual health records, the authors have predicted hypertension with the help of various classification techniques [17]. In [18], hypertension status is classified with classification accuracy of 96.3% using linear discriminant analysis. Alternatively, artificial neural network was used to obtain area under curve of 0.77 [19]. Our study has focused on the development of a robust CAD tool for the assessment of nascent HTN with a greater number of subjects evaluated. The main contribution of the paper is as follows:

- Various transformation techniques, such as discrete wavelet transform, contourlet transform, dual tree complex wavelet transforms, are used to analyze apical 4-chamber view of heart.
- Graph embedding is done using locality sensitive discriminant analysis.
- Dataset which comprises apical 4-chamber normal and HTN images is created.

Our paper is subsequently organized as follows: In Section 2 the collection of ultrasound images utilized is outlined. The framework for the proposed model is described in Section 3. The results and discussion are provided in Sections 4 and 5, respectively. Closing remarks are presented in Section 6.

## 2. Dataset Description

**Figure 1:**
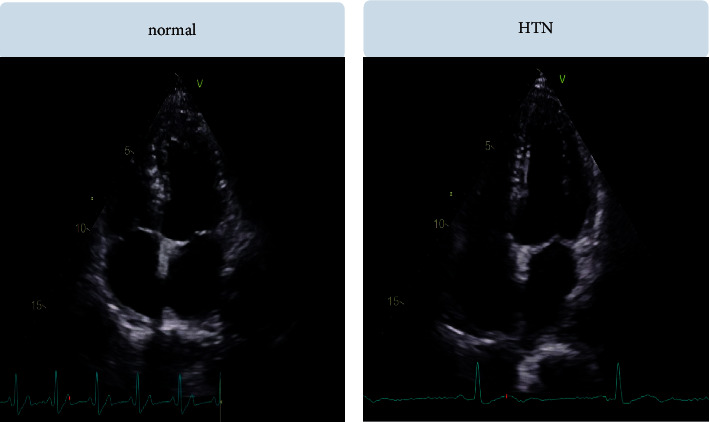
Normal and HTN image samples.

For our study, we collected the data of 54 patients with incidentally detected HTN who visited the cardiology outpatient department for evaluation. Diagnosis was confirmed by the presence of high blood pressure readings obtained from three separate visits. Fifty age-matched healthy individuals who approached the department for a routine health checkup were used as controls. Patients with secondary HTN, renal failure, known ischemic heart disease, congenital heart disease, valvular heart disease with a moderate or greater severity, and/or ejection fraction of less than 55% were excluded from the study. This prospective study was initiated after obtaining institutional ethics committee clearance, and informed consent was acquired from all patients. The study participants underwent complete echocardiographic examination using the Vivid S60 GE healthcare echocardiographic system controlled by an experienced sonographer. Standard apical 4-chamber and 2-chamber views through the probe placed at the apical region, along with the parasternal short axis plane at papillary muscle level, were recorded for further analysis by the CAD system.  Table 1 shows the details of subjects and images used in our study. Sample images used in the present study are provided in Figure 1.

## 3. Proposed Model

**Figure 2:**
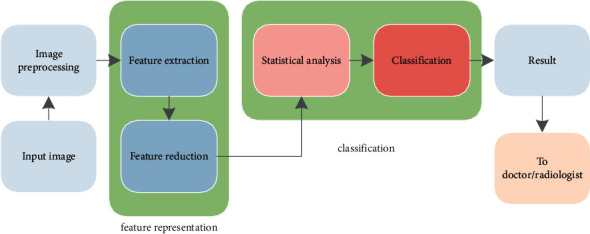
Systematic view of the proposed model.

For the efficient categorization of heart US images, the proposed model consists of two major modules, namely, (i) feature representation and (ii) classification, as shown in Figure 2. Initially, the US images are preprocessed to enhance the performance of the proposed system. All the labels and signal representation in the images (please refer to Figure 1) are removed by generating the mask; as a result, only heart region is extracted. Then, contrast limited adaptive histogram equalization (CLAHE) is applied to enhance the pixel values of the heart images [20]. Thereafter, the preprocessed images are further applied to the aforementioned modules, for proper identification. In feature representation module, the heart images are characterized to know the variations in the pixel arrangement. Further, these features are arranged using their significance level. Finally, various classifiers are used to predict the class labels of the 4-chamber heart images. Details of these modules are presented briefly in the following sections.

### 3.1. Feature Representation

This stage consists of two modules: (i) feature extraction and (ii) feature reduction. Initially, the features are extracted from the US images using various transformation techniques, including the contourlet transform and dual tree complex wavelet transforms. Furthermore, feature size is reduced using an efficient dimensionality reduction technique termed locality sensitive discriminant analysis (LSDA), employed for the efficient representation of the features. These stages are described in the following section.

#### 3.1.1. Feature Extraction Using Transformation Techniques

Feature transformation is a technique which projects the original images into another domain, so that significant details are enhanced. In this study, we have utilized a set of different feature transformation techniques, as follows.

#### 3.1.2. Discrete Wavelet Transform (DWT)

DWT is extensively used in image processing for image compression and feature extraction. It is widely employed to compress still images. DWT compresses the images by decomposing the signal into sub-bands or wavelets. These wavelets can be further represented as a scaled version of a mother wavelet. Therefore, computing the DWT of a signal or image provides a wavelet coefficient and a scaling factor. The mother wavelet is also called the cutting window. This window is convolved with the original signal to obtain the coefficients and factors [21]. In addition to compression, the DWT also makes for smoother color toning and clearer edging at regions with sharp changes of color.

#### 3.1.3. Contourlet Transform (CNTLet Transform)

The CNTLet transform is a directional multi-resolution analysis framework. It is a two-dimensional transform employed for multidimensional image representation. This transformation produces flexible multi-resolution, local, and directional image expansion with contour segments. Its main property is capturing geometric information in the image. In the frequency domain, it provides multiscale and directional decomposition (Do and Vetterli [22]). It works in two stages as a double filter bank construction. In the first stage, the image is decomposed into sub-bands by using Laplacian pyramids (Do and Vetterli [23]). Thereafter, each sub-band is passed through directional filter banks. This provides directionality, anisotropy, multi-resolution, and localization in the transformed image. The CNTLet transform of an image can be represented using fewer coefficients, outperforming other transformation techniques. These coefficients are later input for feature extraction.

#### 3.1.4. Dual Tree Complex Wavelet Transforms (DTcomWT)

DTcomWT is a variant of the discrete wavelet transform. It overcomes the problem of shift variance and the low directional selectivity observed in the discrete wavelet transform. DTcomWT has the following desirable properties: approximate shift invariance, good directional selectivity, perfect reconstruction, limited redundancy, and efficient order-N computation [24]. This technique is widely used for texture characterization [25].

#### 3.1.5. Curvelet Transform (CRVLet Transform)

A CRVLet transform is a multiscale directional transform. This transform was designed to represent the edging on a smooth curve more efficiently, with fewer coefficients and greater accuracy of construction. It is similar to the wavelet transform, with the added property of directional specificity (the orientation parameter), in addition to the dimensionality and locational parameters [26].

#### 3.1.6. Shearlet Transform (SHELet Transform)

The SHELet transform can be described as a directional data approximation scheme. It is widely used to represent multidimensional signals and images. In this transform, a basic wavelet termed the mother SHELet is used to create multiple shearlets. The daughter shearlets are formed by operating the mother shearlet with dilation, shearing, or translation. In certain applications, the shearlet transform is utilized along with a continual transform termed the continual shearlet transform. This transform has the potential to determine geometrical properties such as orientation and curvature of discontinuity. In an advanced technique, which utilizes cone-adapted shearlets, the frequency domain is divided into a low-frequency component and two conic regions, with the frequency coefficients being used for classification [27–29].

#### 3.1.7. Continual Wavelet Transform (CWT)

The CWT is a function of translational and dilation parameters. By varying this parameter, CWT can be used for various applications such as detection of shapes, edges, and contours. This method involves convolution of the analyzing function with the original signal. Using this technique, a signal can be represented as a linear superposition of elementary functions, termed the analyzing wavelets [30].

#### 3.1.8. Empirical Wavelet Transform (EWT)

The EWT technique is employed for signal decomposition. It is an adaptive method for analyzing signals. It is unlike the Fourier transform, which implements a predefined basis function. This method rather constructs a basis function from signal information. It decomposes the signal by frequency. Features are then extracted based on the correntropy taken for each band [31].

#### 3.1.9. Feature Reduction

LSDA is widely used for dimensionality reduction. It is a novel linear discriminant analysis (LDA) method. Like LDA, this method is used to discriminate the data points into different classes. LSDA overcomes the disadvantage of the LDA method, which fails to discover the local geometrical structure of the data. One of the prime properties of LSDA is the preservation of the local geometrical data structure. Therefore, this method is used when there are insufficient training samples. In this case, local structure is more important than global structure [32, 33].

### 3.2. Classification

In this stage, initially all features are tested, using Student's *t*-test to determine significance level. Based on the obtained score, the features are organized. Moreover, labels of these features obtained from the input samples are input to classifiers to distinguish between the two classes. The classifiers used include decision tree (DT) [34], discriminant linear (DL), discriminant quadratic (DQ) [35], and support vector machine (SVM) with different kernels such as radial basis function (RBF); polynomial kernels of order 1, 2, and 3 [36] (i.e., svmRBF, svmPoly_1, svmPoly_2, and svmPoly_3); k-nearest neighbor (kNN) [34]; and probabilistic neural network (probNN) [37]. We have adopted evaluation parameters such as accuracy, positive predictive value (PPV), sensitivity, and specificity to determine the quality of classification. We have used the 10-fold cross-validation approach during training and testing to improve the generality of the proposed model on the analyzed dataset.

## 4. Results

For the purpose of evaluation, 51 images of 51 normal subjects and 61 images of 61 abnormal subjects were initially obtained. These images were preprocessed to remove unwanted information. Initially, adaptive histogram equalization was applied; the images were then resized to 512 × 512 pixels after cropping. Furthermore, various multi-resolution techniques were applied to both the normal and HTN images. The values of the generated features were as follows: DWT: 2560; CNTLet: 4096; DTcomWT: 9216; CRVLet: 1839; SHELet: 15872; CWT: 8192; EWT: 512. Having a large number of features, however, is computationally demanding. To reduce the number of features, locality sensitive discriminant analysis is used, which resulted in 30 features. The training and testing were done based on the 10-fold cross-validation scheme. The average accuracy, sensitivity, positive predictive value (PPV), and specificity show the system performance. The complete model was executed on a MATLAB platform with standalone computer system. The obtained results for different multi-resolution analysis methods are shown in Table 2. It is noted that SHELet features are efficient and produce stable results with various classifiers.

## 5. Discussion

HTN is a major cause of cardiovascular disease; therefore, early detection can reduce morbidity and mortality. In this work, we have developed a CAD tool for the extraction of features useful for HTN characterization and detection of the disease. After preprocessing of the image to remove noise, features were extracted. Various mathematical tools were used for this purpose. The DWT was incorporated in our study, and this technique is also widely used for feature extraction elsewhere. A variant of DWT termed the dual tree wavelet transform, possessing the desirable properties of approximate shift invariance, good directional selectivity, quality reconstruction, limited redundancy, and efficient order-N computation, was also employed. Certain finer details in the image, including smoothly varying edging, levels, and curves, can be readily captured using the contourlet and curvelet transforms. The SHELet and CWT transform highly perform and can determine geometrical properties such as orientation, curvature of discontinuity, shape, edging, and contouring.

**Figure 3:**
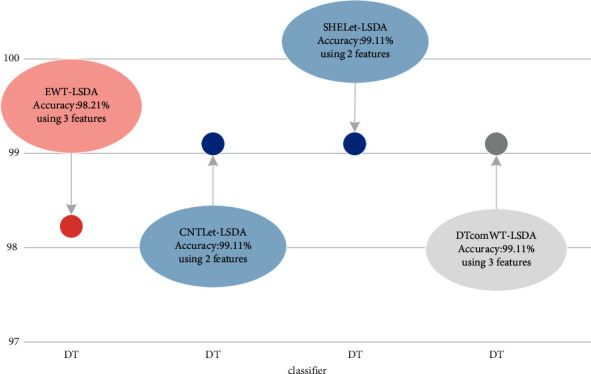
Best performance of various multi-resolution approaches.

All of these transforms produce a large number of features. Processing a large number of features is tedious and time-consuming. Therefore, it is important to effectively reduce the feature set. For this purpose, a dimensionality reduction tool was used, namely, LSDA. One of the prime properties of LSDA is the preservation of local geometrical data structure and effective discrimination. Though several advantages are offered by the DWT and CWT in various applications, it is noted that DWT-LSDA and CWT-LSDA produce overfitting of the distribution. The combination of various methods and their accuracy is shown in Figure 3. It is observed that the DT classifier outperforms other classifiers. The CNTLet-LSDA and SHELet-LSDA methods reached a remarkable accuracy level with the help of dual features. It is evident that the DWT and CWT features produce results which have a negative impact on the new data, due to overfitting [38].

**Figure 4:**
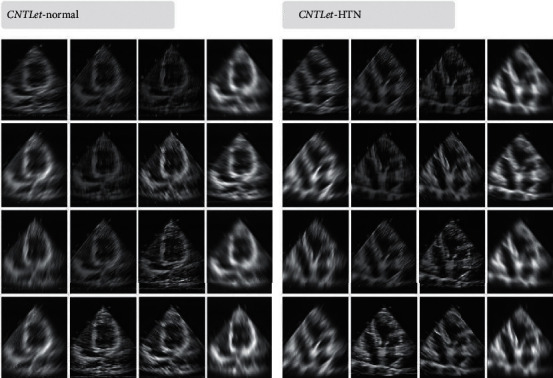
CNTLet transformation for normal and HTN images.

**Figure 5:**
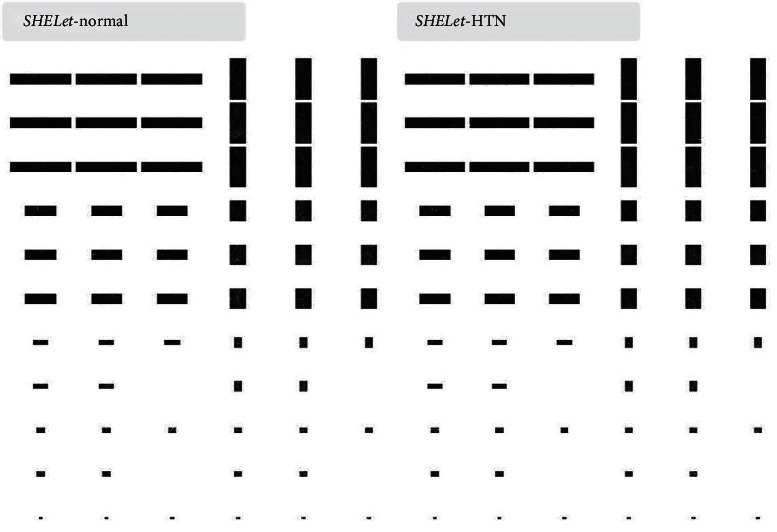
SHELet transformation for normal and HTN images.

Figures 4 and 5 show the CNTLet and SHELet transform images, and there is generation of highly significant features to discriminate normal versus HTN images.

It is noted that the mean and standard deviation (SD) of these transformed LSDA features spread separately, confirming the nonlinearity of the feature handling capability of the system (refer to Tables 3 and 4). The initial 10 features are shown in Tables 3 and 4, and the maximum accuracy obtained by two features is 99.11%, which exhibits a higher significance level.

**Figure 6:**
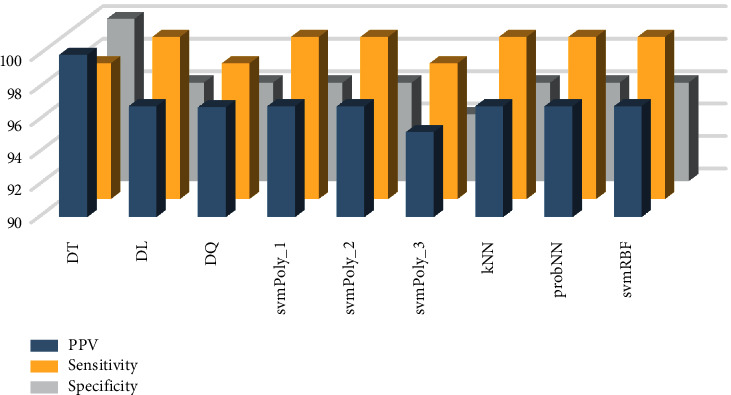
Various classifier performances for CNTLet-LSDA combination.

**Figure 7:**
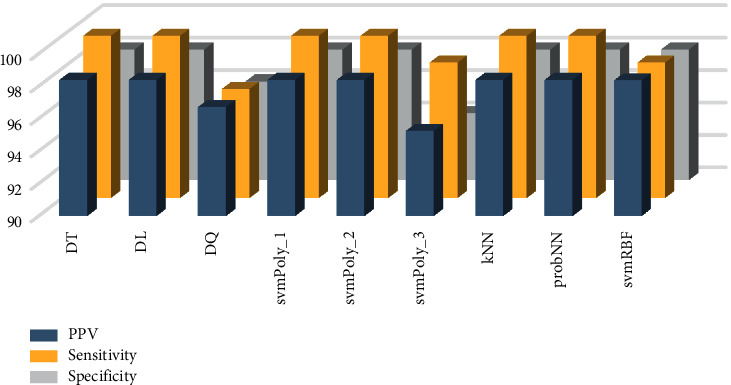
Various classifier performances for SHELet-LSDA combination.

The other evaluating indices for various classifiers using the CNTLet-LSDA and SHELet-LSDA approaches are shown in Figures 6 and 7. It is observed that CNTLet-LSDA reached a specificity of 100% and SHELet-LSDA reached a sensitivity of 100%. Furthermore, the hybridization of these methods helps us to identify the true negative and true positive cases accurately. The experimental results show the efficiency of the system. Further, it can be used by doctors in hospitals or polyclinics to conclude their findings. Hence, the positive effect of the proposed system shows the potentially usefulness in decision making. Hence, it can be used to control and detect hypertensive patients.

The major advantages of the proposed model are listed as follows:

- It is fast and produces an accurate result.
- It requires only two features to predict the input heart image. Hence, a less configured embedded system is required to install it in hospital settings.
- It is a generalized model and can be applied to other modalities, though this will require further investigation.

The proposed system is developed using smaller dataset, and in future we want to generalize the system using more number of images. In addition, we plan to develop the system which can categorize and analyze various heart diseases using apical 4-chamber heart view.

**Figure 8:**
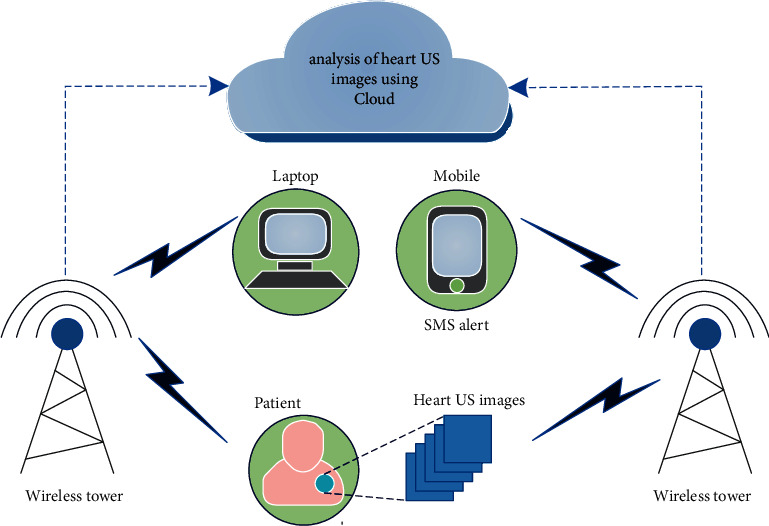
Wireless based IoT architecture for the assessment of cardiac structural alteration in hypertension.

The proposed system can be used to give diagnostic results to doctors and patients by using wireless based Internet of Things (IoT) architecture as shown in Figure 8. The heart ultrasound images of patients to be tested are sent to the cloud where our trained model is kept. The cloud based server is connected to doctors and patients through a wireless network, to evaluate the cardiac structural alteration in the ultrasound images. Such wireless based systems will send a report to the patients' mobile whereas IoT based system will send clinical decisions to doctors to detect the HTN immediately and accurately. Furthermore, the test images can be used to train the model and make it more robust. These models can be further developed using hybrid techniques to achieve efficacy in performance measures. In addition, this system can be deployed to rural area, where there is a scarcity of heart specialists. Based on the results, patients can undergo further treatment. Hence, the patient health management system can be improved.

## 6. Conclusion

It would be useful to predict the nature of cardiovascular diseases in order to improve the public health, and in this paper, we present a novel and efficient tool to forecast HTN-induced cardiac structural alteration using ultrasound images. The experimental results show that the system achieved a level of 100% sensitivity and specificity for two different feature sets. Since the system utilizes a dataset of limited size, in future work, we would like to further elaborate its effectiveness with a larger population. Use of deep models and a hybrid feature learning module could be assistive to train this dataset. It could then be utilized more effectively in the future, for clinical diagnostics.

## Figures and Tables

**Table 1:** Data description.

Classes	No. of subjects	Male/female count	Age range (mean ± SD)	No. of images
Normal	51	31/19	52.59 ± 17.79	51
HTN	61	42/12	49.36 ± 12.74	61

**Table 2:** Classification accuracy (%) for various classifiers using different multi-resolution techniques.

Classifier	CNTLet	CRVLet	DTcomWT	EWT	SHELet	
DT	99.11	97.32	99.11	98.21		99.11
DL	98.21	97.32	98.21	98.21		99.11
DQ	97.32	96.43	95.54	89.29		96.43
svmPoly_1	98.21	97.32	99.11	98.21		99.11
svmPoly_2	98.21	97.32	98.21	97.32		99.11
svmPoly_3	96.43	96.43	97.32	94.64		96.43
svmRBF	98.21	97.32	98.21	98.21		98.21
kNN	98.21	97.32	98.21	97.32		99.11
probNN	98.21	97.32	99.11	98.21		99.11

**Table 3:** Initial 10 features of CNTLet transformed features.

Features	Normal		HTN		*p* value	*t* value
	Mean	SD	Mean	SD		
LSDA4	0.0031	0.0018	0.0005	0.0012	≤0.001	8.9935
LSDA3	−0.0106	0.0017	−0.0085	0.0014	≤0.001	7.1636
LSDA2	0.0102	0.0028	0.0098	0.0001	0.2577	1.1449
LSDA13	−0.0054	0.0034	−0.0060	0.0030	0.3231	0.9930
LSDA1	0.0120	0.0031	0.0116	0.0001	0.3292	0.9852
LSDA5	0.0040	0.0027	0.0036	0.0011	0.4096	0.8301
LSDA10	−0.0141	0.0022	−0.0138	0.0018	0.4429	0.7705
LSDA16	−0.0023	0.0030	−0.0027	0.0018	0.4886	0.6958
LSDA23	0.0184	0.0011	0.0182	0.0029	0.5616	0.5828
LSDA12	0.0170	0.0028	0.0172	0.0018	0.6112	0.5103

**Table 4:** Initial 10 features of SHELet transformed features.

Features	Normal		HTN		*p* value	*t* value
	Mean	SD	Mean	SD		
LSDA2	−0.0363	0.0388	0.0457	0.0467	≤0.001	10.1429
LSDA5	0.0228	0.0771	−0.0091	0.0425	0.0101	2.6400
LSDA3	0.0210	0.0311	−0.0049	0.0740	0.0148	2.4877
LSDA1	−0.0058	0.0896	0.0107	0.0074	0.1975	1.3058
LSDA4	−0.0034	0.0465	−0.0155	0.0723	0.2870	1.0703
LSDA8	−0.0086	0.0771	−0.0006	0.0524	0.5325	0.6268
LSDA7	0.0058	0.0740	−0.0012	0.0520	0.5705	0.5695
LSDA6	0.0103	0.0177	0.0164	0.0860	0.5867	0.5463
LSDA30	−0.0007	0.0716	0.0053	0.0588	0.6342	0.4774
LSDA24	0.0153	0.0663	0.0095	0.0646	0.6397	0.4694

## Data Availability

No data were used to support this study.
